# Predictors of Bribe-Taking: The Role of Bribe Size and Personality

**DOI:** 10.3389/fpsyg.2018.01511

**Published:** 2018-09-10

**Authors:** Marek A. Vranka, Štěpán Bahník

**Affiliations:** Faculty of Business Administration, University of Economics, Prague, Czechia

**Keywords:** morality, corruption, bribe-taking, HEXACO, reaction times

## Abstract

Laboratory studies allow studying the predictors of bribe-taking in a controlled setting. However, presently used laboratory tasks often lack any connection to norm violation or invite participants to role-play. A new experimental task for studying the decision to take a bribe was designed in this study to overcome these problems by embedding the opportunity for bribe-taking in an unrelated task that participants perform. Using this new experimental task, we found that refraining from harming a third party by taking a bribe was associated with lower offered bribes and higher scores of the participants on the honesty-humility scale from the HEXACO personality inventory. A trial-level analysis showed that response times were longer for trials with bribes and even longer for trials in which bribes were accepted. These results suggest that taking a bribe may require overcoming automatic honest response and support the validity of the honesty-humility scale in predicting moral behavior.

## Introduction

Corruption a considerable negative impact on economic and social development (Jain, 2001). Since bribery is an illegal and concealed activity, the research on bribery at the individual level presents a severe challenge. A potential solution of this problem has been recently offered by laboratory experiments studying dishonest behavior in general (Mazar and Ariely, 2006; Rosenbaum et al., 2014) and corruption in particular (Abbink, 2006; Köbis et al., 2015). Using an experimental approach, individual behavior can be observed in model laboratory situations designed in a way that allows individuals to act corruptly (e.g., to take bribes) under conditions analogous to conditions in the real world. Moreover, the controlled nature of the laboratory environment allows researchers to easily model various policies and interventions and observe their effect on the prevalence of bribe-taking (Abbink, 2006). Even though all laboratory studies can be criticized because of their artificiality, current findings support the external validity of laboratory studies of dishonesty in general (Potters and Stoop, 2016; Dai et al., 2017) as well as corruption in particular (Armantier and Bóly, 2012). The potential of this approach is illustrated by a growing number of published experimental studies on corruption (for reviews, see Abbink and Serra, 2012; Banuri and Eckel, 2012; Bobkova and Egbert, 2013).

The majority of existing studies model corruption using a modified version of the “Trust game” in which a player in the role of a company or a citizen can transfer funds to a second player, usually called “public official.” The public official can then reciprocate this “bribe” by increasing the final reward of the first player at the expense of other players – usually representing the public (e.g., Abbink et al., 2002; Alatas et al., 2009a,b; Cameron et al., 2009; Barr and Serra, 2010; Drugov et al., 2014; D’Adda et al., 2016; Fišar et al., 2016). The presence of an explicit decision whether to harm someone to gain a financial benefit differentiates experiments studying corruption from similar studies focused on dishonesty in general. In some cases, sending a bribe is associated with a transaction cost, regardless of whether it is accepted or not, and there can also be a chance that the corrupt behavior is discovered and punished (e.g., Abbink et al., 2002; van Veldhuizen, 2013).

Some existing studies use neutral language in the instructions, whereas others explicitly mention corruption and bribes. Explicitly mentioning bribes was shown to decrease the prevalence of corrupt behavior (Barr and Serra, 2009), probably because immorality of “corrupt” behavior may not be evident when instructions employ neutral language (Abbink, 2004). On the other hand, when the word corruption is used, participants always have to engage in role-playing. Either they pretend that they themselves are in the described scenario and behave according to their own moral compass, or they pretend to play a role introduced in the scenario and behave according to the moral norms they consider appropriate for the role. They may even act more corruptly than they would in real life, because they expect that a firm or a public official would act corruptly in the given situation. This presents a serious problem for any experimenter; either the experimental task lacks important features of the studied behavior (namely, the connection to moral norms) or the studied behavior depends in large part on an idiosyncratic interpretation of experimental stimuli (Levitt and List, 2007).

In the present study, we introduce a new experimental task that overcomes this problem, because in the task, taking a bribe is always clearly associated with a norm violation, even when neutral language is employed. Moreover, unlike the tasks used in previous studies, in our new task participants face many opportunities to take bribes of various sizes as well as trials without any bribes. This allows us to examine how bribe-taking develops during the task, as well as what factors affect the length of decision whether to take a bribe or not. At the beginning of the task, participants are instructed to perform the task according to simple rules. For their work, they receive a fixed payment each time they perform the task, regardless of whether they do it in accordance with the rules or not – as do many public officials and employees in general. However, when they perform the task in violation of the given rules, another party is harmed. In the described setting, participants are motivated to perform their task correctly, based on the assumption that they do not want to deliberately harm the other party. The relevant behavioral norms follow from the rules of the task that participants are asked to perform, and there is, therefore, no need for role-playing. A bribe is introduced seamlessly into this setting as an offer of a financial reward for violating the rules. Participants can thus earn an additional reward (i.e., the bribe) by performing the task in violation of the rules while causing harm to the other party. Alternatively, participants can pass on this opportunity and simply perform the task according to the instructions. For simplicity, we focus solely on the behavior of the bribee in the current study. No participant is assigned the role of a briber, and therefore there is no possibility to study the decision to offer a bribe or the development of a relationship between briber and bribee. However, these aspects can be added to the game relatively easily in future implementations. Moreover, their current absence does not preclude us from studying a bribee’s decision-making in general, as there are also real-life instances of corruption in which an official is offered bribes without knowing the bribers and without the need to interact with them any further – such as when a bribe is included inside an application or together with documents the official is supposed to check.

The structure of the described experimental task in a simplified manner captures the various characteristics of bureaucratic work such as repetitive tasks, fixed payment, little to no quality control, straightforward rules of conduct, and opportunities for accepting bribes. At the same time, the description of the game and its rules can be kept abstract and neutral, without any references to public officials and bribery.

In the remainder of the paper, we describe in detail the new experimental task and present the results of its implementation. To demonstrate support for the validity of the new task for studying bribe-taking, we first show that participants are more likely to break the rules of the game when offered a bribe and that they are even more likely to do so in the case of larger bribes. Furthermore, we show that the perception of accepting a bribe as immoral and rejecting it as moral is associated with a lower likelihood of taking the bribe. We also conceptually replicate some previously found associations between gender, personality traits, and corrupt behavior using the new task. In particular, many previous studies found that women tend to act less dishonestly than men in general (Ward and Beck, 1990) as well as less corruptly in particular (Dollar et al., 2001; Chaudhuri, 2012; Fišar et al., 2016). We observe the same pattern of behavior in our task. In accordance with the results of previous studies showing the association between the honesty-humility trait and fair behavior in economic games (Hilbig and Zettler, 2009, 2015; Hilbig et al., 2015), we show that the honesty-humility scale is negatively associated with corrupt behavior, and this further supports the validity of the task.

Afterward, we take advantage of the fact that our task offers participants many opportunities to take or decline bribes of different sizes during the experiment and we test a hypothesis about moral self-licensing and corrupt behavior. According to the moral licensing theory (Blanken et al., 2015; Effron and Conway, 2015), people who behave morally in one instance feel entitled to behave immorally later, as if they had earned a “license” to do so owing to their previous good deeds. Moral licensing was not previously explored in the context of corruption despite its potentially important implications for real-life anticorruption interventions. We test whether participants who are offered small bribes, which they are likely to reject, will be more likely to accept subsequent larger bribes, having earned a moral license to take bribes after rejecting the smaller bribes.

Finally, we attempt to contribute to the ongoing debate about whether people are intuitively honest or whether they need deliberation to overcome impulses to act dishonestly when given a chance (Weaver et al., 2014), by analyzing the decision times in trials in which participants accept or reject bribes. Despite the initial evidence that people intuitively act selflessly when given a chance to profit at the expense of others (Rand et al., 2012), these results were not obtained in a recent large-scale replication attempt (Bouwmeester et al., 2017). While previous studies usually employed one-shot interactions, our new task allows us to explore possible interactions of reaction times with the number of trials, size of a bribe, and cumulative harm caused to a third party.

## Materials and Methods

Materials, data, the R script used for analysis, and preregistration of study hypotheses can be found at https://osf.io/ak8un/.

### Participants, Procedure, and Materials

We collected data from 200 participants (73% female, median age = 23 years, 84% university students) recruited from the participant pool of our experimental laboratory. The experiment was conducted as the second part of a larger set of unrelated studies and took approximately 50 min. All participants were compensated for their participation in the whole set of studies with 100 CZK (∼4 USD). Moreover, they could gain additional money in the experiment, as described in more detail below. The experiment was conducted on computers using a custom-written Python program. The data collection took place in a laboratory with groups of up to 13 people seated at workstations separated by dividers. The whole experiment was conducted in Czech. Participants gave an informed consent at the beginning of the experiment and they were debriefed regarding the purpose of the research at its end.

Participants were asked to sort by color 100 objects moving one by one from the left to the right on computer screens. Participants had maximum of 3 s for classifying each object. The objects were combinations of three shapes and three colors (e.g., blue circle, yellow square, orange triangle, and so on). The sorting was done by pressing one of three buttons on a keyboard (“1,” “2,” and “3” on the numerical part of the keyboard). Each of the three buttons was randomly assigned to a combination of color and shape. The assignment changed after each classification. For each sorted object (that is, even incorrectly sorted), participants received a fixed amount of 3 points. At the beginning of the task, 2000 points were assigned to a well-known Czech charitable organization providing humanitarian aid. When the object was not classified to the corresponding *color*, 200 points were subtracted from the points assigned to the charity. With a probability of 0.2, a number (henceforth called “bribe” for simplicity) was written on an object. When such an object was sorted to the corresponding *shape*, the participant gained the number of points equal to the written number. Because the buttons were assigned to random combinations of the three colors and shapes on each trial, it was possible that in some trials the classification according to color was the same as the classification according to shape. In these trials, participants gained points for the bribe even without causing any harm to the charity. Owing to this feature of the task, it was not possible to directly estimate who was acting dishonestly just from the final amount of participant’s points. Therefore, participants did not need to worry that the experimenter would know that they cheated when they were claiming the money at the end of the experiment.

To test the moral licensing effect, participants were divided into two groups with differing distributions of bribes. In the *low bribes condition*, the bribe value was randomly sampled from values 10, 30, 50, 80, 170, 190, 200, and 300. In the *high bribes condition*, the bribe value was randomly sampled from values 100, 110, 130, 150, 170, 190, 200, and 300. Only the values of the four lowest bribes were different between the two conditions. Participants in the low bribes condition were thus offered more bribes that they would be likely to reject. According to the moral licensing theory, this should, in turn, make them more likely to accept the larger bribes in comparison to participants in the high bribe condition.

**FIGURE 1:**
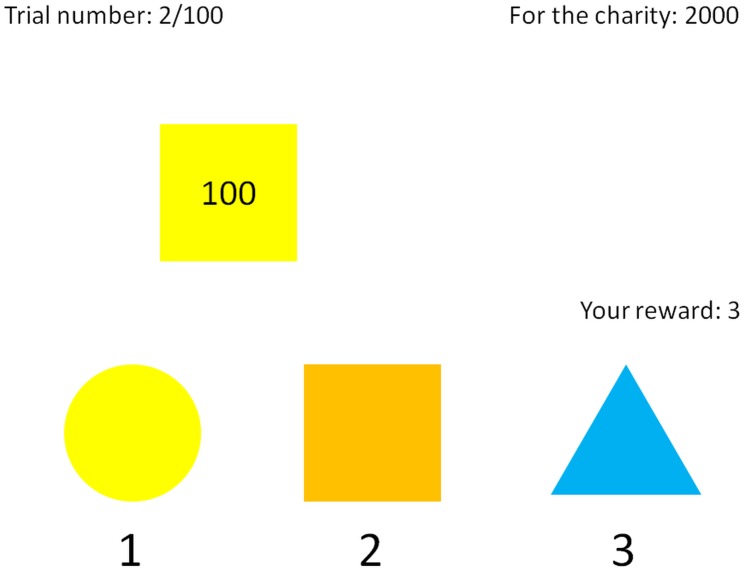
An illustration of a computer screen seen by a participant. The top row shows information about the number of the current trial, the total number of trials, and the number of points currently assigned to the charity organization. In the middle of the screen, an object (a yellow square in this case) is moving from the left side of the screen to the right. The current participant’s reward in points is shown to the right of the screen. In the bottom row, a participant sees which shapes and colors are assigned to keys “1,” “2,” and “3” in this trial (in this example, 1 is yellow circle, 2 is orange square, and 3 is blue triangle). If the participant presses “1,” the object would be sorted by its color, that is correctly, and the participant would gain 3 points. If the participant presses “2,” the object would be matched to a wrong color, and would cause a loss of 200 points for the charity, but it would be sorted according to its shape, allowing the participant to gain the 100 points marked on the object in addition to the 3 points awarded for each sorted object.

The information about the number of the current trial and the points gained for oneself and the charity was displayed during the whole trial (see **Figure 1** for an illustration of a computer screen seen by a participant). We fully explained the task to the participants before the experiment began. We did not provide the information about the probability of the bribe and the distribution of its values. Participants had a 1/13 chance that the points they gained during the experiment would be converted to a monetary reward for themselves and the charity (using the conversion rate 10 points = 1 CZK). The money gained for the charity was summed after the data collection was completed and transferred to its bank account. At the end of the whole set of studies, participants filled the HEXACO personality questionnaire (Lee and Ashton, 2004) that measures the same five personality dimensions as the NEO personality inventory (McCrae and Costa, 1997) with an addition of the sixth dimension of honesty-humility. We also asked the participants about their belief of the purpose of the study and they stated, using a scale ranging from “1 – certainly no” to “4 – certainly yes” whether they consider performing a misclassification to get a bribe as despicable, dishonest, unjust, or immoral and whether they consider performing a correct classification to not cause a loss to the charity as just, praiseworthy, honest, or moral.

## Results

^1^Based on the preregistered exclusion criteria, we excluded the trials without any correct classification (either by shape or color) from the analysis (2.13%). We also excluded three participants who had more than 10 excluded trials. We conducted all analyses with 19,574 observations from the remaining 197 participants.

### Task Performance

In total, participants classified 94.9% of the trials correctly according to color. Participants therefore classified most of the objects according to the instructions. When an object was classified according to shape (as was the case in 38.9% of the trials), in most cases it was in a situation where classifications according to color and shape were aligned; 33.8% of trials were classified correctly according to both criteria. When the classification criteria were misaligned, and participants were offered a bribe, they took it in 15.1% percent of cases.

Thirty-nine percent of participants classified all the objects according to color and thus did not lose any of the 2000 points for the charity. The distribution of the final outcome for the charity was highly negatively skewed (skewness = −3.29) with a mean of 936 points (*SD* = 2029) and a median of 1800 points (*IQR* = 1200). Only a minority of participants had the final outcome for the charity negative (13.7%) or zero (1.5%). On average, participants earned 1598 points for themselves (*SD* = 665, *Mdn* = 1510, *IQR* = 820). Earnings for oneself and the charity were negatively correlated, *r*_S_ = −0.52, 95% CI = [−0.62, −0.40]. Earnings for the charity strongly negatively correlated with the proportion of bribes taken in the trials in which the classification according to shape and color was misaligned (*r*_S_ = −0.85, 95% CI = [−0.89, −0.80]). The correlation of the proportion of bribes taken in the trials in which the classification according to shape and color was misaligned with the reward for a participant was positive and also significant (*r*_S_ = 0.57, 95% CI = [0.46, 0.66]). Given that the final outcome for the charity is influenced by participants’ mistakes, we use only the proportion of bribes taken and the final participants’ earnings in later analysis. While the proportion of bribes taken does not take into account the size of bribes, final participants’ earnings are influenced also by “bribes” in trials in which the sorting criteria were not misaligned.

### Task Perception

**FIGURE 2:**
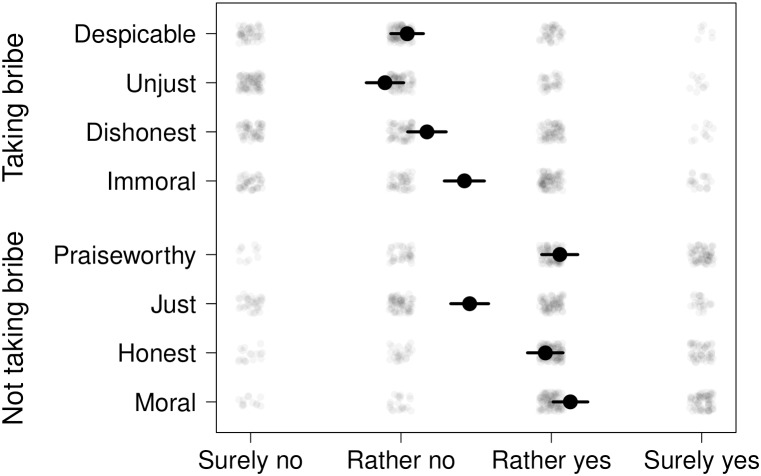
Perception of the task by participants. The answers were coded on a 4-point scale and their means are shown by black points. Error bars represent 95% confidence intervals. The transparent points display individual answers.

After the experiment, we asked the participants about their perceptions of sorting the objects according to color or shape in cases where the object was associated with a bribe. On a scale from one to four (1 – certainly not, 2 – rather not, 3 – rather yes, 4 – certainly yes), participants did not generally perceive taking the bribe as despicable (*M* = 2.04), dishonest (*M* = 2.17), and unjust (*M* = 1.89), and they were ambiguous about whether it is immoral (*M* = 2.42). Similarly, they were ambiguous about whether not taking the bribe is just (*M* = 2.46). However, they mostly viewed not taking the bribe as praiseworthy (*M* = 3.06), honest (*M* = 2.96), and moral (*M* = 3.13) (see **Figure 2**). For the purpose of further analysis, we computed a composite score of task perception for each participant by averaging the eight ratings (*M* = 2.5, *SD* = 0.56). The composite *task perception* rating showed reasonable internal consistency (Cronbach’s α = 0.80, 95% CI = [0.73, 0.87]).

### Participant-Level Analysis

We found a predicted association between the HEXACO honesty-humility scale and the final outcome from the task. Participants scoring higher on the honesty-humility scale had a lower final reward for themselves (*r*_S_ = −0.25, 95% CI = [−0.38, −0.11], *p* < 0.001), and took a lower proportion of bribes in the trials in which the classification according to shape and color was misaligned (*r*_S_ = −0.15, 95% CI = [−0.29, −0.00], *p* = 0.03).^2,3^ The other five personality traits measured by HEXACO were not significantly associated with the final outcome. The composite score of task perception was negatively – but not significantly – associated with participants’ earnings (*r*_S_ = −0.12, 95% CI = [−0.26, 0.03], *p* = 0.10), and significantly associated with the proportion of bribes taken (*r*_S_ = −0.24, 95% CI = [−0.37, −0.11], *p* < 0.001). We found that women had on average a lower reward for themselves (*r*_S_ = −0.17, 95% CI = [−0.31, −0.03], *p* = 0.02), and lower proportion of bribes taken (*r*_S_ = −0.15, 95% CI = [−0.30, 0.00], *p* = 0.03) than men. As expected, participants in the low bribe condition earned less for themselves than participants in the high bribe condition (*r*_S_ = −0.20, 95% CI = [−0.33, −0.06], *p* = 0.005, *Mdn*_low_ = 1420, *Mdn*_high_ = 1600); however, there was no significant difference in the earnings for the charity between the two conditions (*r*_S_ = 0.03, 95% CI = [−0.12, 0.17], *p* = 0.67, *Mdn*_low_ = 1800, *Mdn*_high_ = 1800), and no significant difference in the proportion of bribes taken (*r*_S_ = 0.04, 95% CI = [−0.10, 0.18], *p* = 0.59).

### Trial-Level Analysis

We used mixed-effect logistic regression for analyzing bribe-taking on a trial level and mixed-effect linear regression for analyzing reaction times (Gelman and Hill, 2006; Baayen et al., 2008). We rescaled all the predictors to a −0.5 to 0.5 range to simplify the interpretation of the estimated parameters.^4^ We always included random intercepts for participants in the model. Random slopes for participants differed between models, and hence variables for which we included random slopes are described with the models.

#### Effect of Bribes

To assess the effect of bribes on classification, we performed an analysis with all trials that were classified correctly according to one of the two criteria (shape and color) excluding the trials in which both criteria were aligned. Classification based on shape (i.e., corresponding to taking a bribe when it was present) was used as the dependent variable and the presence of a bribe and the trial order as well as their interaction as predictors. We found out that the data were autocorrelated and hence we also included the classification on the previous trial as a covariate. This also means that the first trial was not included in the analysis. The partial autocorrelation coefficients were not significantly different from zero for higher trial lags. We included participant random slopes for both predictors. Participants seemed to be less likely to classify objects according to shape in later trials (*z* = −1.81, *p* = 0.07, *OR*^5^ = 0.38, 95% CI = [0.13, 1.08]), and more likely if the object was associated with a bribe (*z* = 3.54, *p* < 0.001, *OR* = 4.03, 95% CI = [1.86, 8.74]). The response on a previous trial also predicted the subsequent response (*z* = 4.36, *p* < 0.001, *OR* = 1.54, 95% CI = [1.27, 1.87]). Furthermore, the interaction of the trial order and the presence of a bribe was significant (*z* = 5.28, *p* < 0.001, ratio of *OR* = 11.68, 95% CI = [4.69, 29.06]), showing that the effect of a bribe on the classification of an object according to its shape was larger in later trials.

#### Effect of a Bribe Size

**FIGURE 3:**
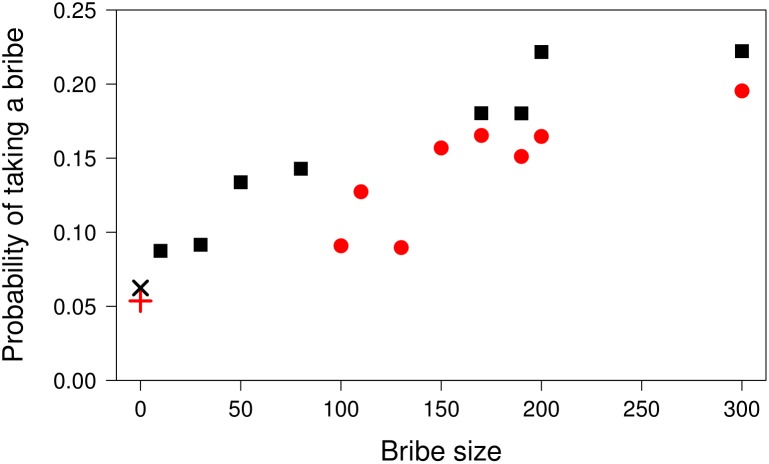
The effect of bribe size on the probability of taking the bribe. The figure shows the average probability of taking a bribe in trials with misaligned classification criteria depending on the bribe size. The displayed points are computed across all participants and trials separately for the low bribe condition (black squares and saltire) and high bribe condition (red dots and cross). It is possible to see that the probability of taking a bribe increases with larger bribes and that participants are more likely to classify an object according to its shape when a bribe was present (squares and dots) than when it was absent (saltire and cross). Note that the abscissa ends at the probability of 0.25, not 1.

Next, we repeated the analysis using trials with misaligned criteria but including only trials with bribes. That is, the analysis was performed only for the trials in which participants had to decide between taking a bribe and leaving it. We included trial order and bribe size and their interaction as predictors and the main effects also as random slopes for participants. Participants were less likely to take the bribe in later trials (*z* = −2.15, *p* = 0.03, *OR* = 0.14, 95% CI = [0.02, 0.84]) and more likely to take larger bribes (*z* = 3.10, *p* = 0.002, *OR* = 13.19, 95% CI = [2.58, 67.48]) (see **Figure 3**). The interaction of the two factors was not significant (*z* = 1.62, *p* = 0.10, *OR* = 7.28, 95% CI = [0.66, 80.28]). The response on a previous trial was again associated with the response on the subsequent trial (*z* = 1.93, *p* = 0.05, *OR* = 1.41, 95% CI = [0.99, 1.99]). When gender and honesty-humility were added in the model, higher honesty-humility was associated with a lower probability of taking a bribe (*z* = −1.71, *p* = 0.09, *OR* = 0.52, 95% CI = [0.24, 1.10]), and females were less likely to take a bribe (*z* = −1.94, *p* = 0.05, *OR* = 0.33, 95% CI = [0.11, 1.01]), but neither of the effects was significant.

#### Low/High Bribe Condition Effect

Since the two conditions differed in their bribe sizes, we conducted the analysis testing the effect of the condition using only the larger half of bribes that was held constant between the two groups. We included only random slopes for the trial order owing to convergence issues.^6^ The effect of the condition was not significant (*z* = −0.06, *p* = 0.95, *OR* = 0.96, 95% CI = [0.23, 4.00]). Larger bribe sizes were associated with a higher probability of the participants taking them even on this reduced range of bribe sizes (*z* = 2.89, *p* = 0.004, *OR* = 8.28, 95% CI = [1.97, 34.79]), but the order effect was no longer significant (*z* = −1.23, *p* = 0.22, *OR* = 0.14, 95% CI = [0.01, 3.18]). The response on a previous trial was again associated with the response of the subsequent trial (*z* = 2.57, *p* = 0.01, *OR* = 1.86, 95% CI = [1.16, 3.00]).

#### Response Times

Finally, we conducted an analysis of log-transformed response times. The distribution of response times (*Mdn* = 894 ms, *IQR* = 426 ms) was positively skewed (skewness = 1.43); hence, we used logarithmic transformation for the analysis of reaction times to make their distribution closer to normal. We also excluded one outlying trial with a 32 ms reaction time. Since reaction time data are usually autocorrelated, we computed partial autocorrelations of log-transformed reaction times for each participant.^7^ The average partial autocorrelation was significantly greater than zero up to the lag of seven trials. We, therefore, included reaction times in the last seven trials as covariates in the analysis to account for the autocorrelation (Baayen and Milin, 2010). We do not report these covariates further, but all were significant in the model. We included in the model random slopes for participants for the presence of bribe, bribe size, misalignment of shape and color criteria, and trial order.

Participants classified the objects faster in later trials {*t*(185.0) = −4.05, *p* < 0.001, *b* = −0.039, 95% CI = [−0.057, −0.020]}. The presence of bribes increased response times {*t*(249.9) = 10.16, *p* < 0.001, *b* = 0.118, 95% CI = [0.096, 0.141]}, and larger bribes increased response times more {*t*(166.0) = 5.17, *p* < 0.001, *b* = 0.085, 95% CI = [0.053, 0.117]}. Participants responded slower on trials in which the color and shape criteria were misaligned {*t*(289.5) = 11.95, *p* < 0.001, *b* = 0.071, 95% CI = [0.059, 0.082]}, but the interaction of the alignment with the presence of a bribe was not significant {*t*(16903.0) = 0.67, *p* = 0.51, *b* = 0.005, 95% CI = [−0.011, 0.021]}. The speed of a response also did not differ based on the total charity earnings at the point of the decision {*t*(194.5) = 0.32, *p* = 0.75, *b* = 0.014, 95% CI = [−0.070, 0.097]}.

Next, we performed an analysis of response times using only the trials in which bribes were offered and taking them was associated with a loss to the charity. We used only the significant predictors from the previous model and added a response on a given trial as another predictor. Even for the selected trials, participants classified the objects faster in later trials {*t*(165.3) = −3.37, *p* < 0.001, *b* = −0.033, 95% CI = [−0.052, −0.014]}, and larger bribes were associated with slower response times {*t*(185.1) = 13.43, *p* < 0.001, *b* = 0.228, 95% CI = [0.195, 0.262]}. Charity earnings at the point of the decision were negatively associated with response times {*t*(164.6) = −4.45, *p* < 0.001, *b* = −0.192, 95% CI = [−0.276, −0.107]}. Taking a bribe was slower than leaving it {*t*(105.1) = 14.34, *p* < 0.001, *b* = 0.278, 95% CI = [0.240, 0.316]}. We then added the interaction between bribe size and the acceptance of the bribe in the model to test whether the effect of bribe size was driven only by rejected bribes. The interaction was not significant, suggesting that the effect of bribe size does not differ between accepted and rejected bribes {*t*(2279.5) = 0.85, *p* = 0.40, *b* = 0.028, 95% CI = [−0.037, 0.093]}. The association of slower responses with higher bribe sizes is also present for participants who did not take any bribe in the selected trials {*t*(71.8) = 7.32, *p* < 0.001, *b* = 0.162, 95% CI = [0.119, 0.206]}, further suggesting that the effect is not driven by previous experience with accepting bribes.

## Discussion

In the present study, we introduced and attempted to validate a new laboratory task for studying a participant’s decision to take a bribe. The new task was created to overcome the limitations of tasks commonly used in previous studies. One limitation of these tasks lies in the fact that they model corruption in a way in which the corrupt behavior itself does not violate any obvious norms of conduct. If the task is then described using purely neutral language, it models corruption and bribe-taking only as simple economic transactions in which another party can be harmed, as in the well-known Dictator or Ultimatum games (Camerer and Thaler, 1995). However, an important psychological characteristic of accepting bribes – namely, the psychological cost of norm violation – is absent as a result. This problem cannot be easily overcome by simply framing the experimental task in terms explicitly related to bribery because this would invite participants to role-play as corrupt public or company officials and would presumably lower the psychological costs of corrupt behavior. Our new task overcomes the above-mentioned problem; the main feature of the experimental task is that the decision to accept or reject bribes is embedded in a setting in which participants are asked to perform an action according to given rules and corrupt behavior is associated with breaking these rules. Moreover, while the tasks in previous studies usually focus on single one-time decisions, our new task consists of many trials, some with different-sized bribes and some with no bribes offered at all. This made studying the development of bribe-taking over a longer period and analyzing decision times associated with accepting and rejecting bribes of various sizes possible, unlike in the case of previously used tasks.

The participants in our study clearly understood the instructions, as is evident from the fact that they correctly classified objects in almost 95% of the trials. That the incorrect classification occurred only in 5% of trials may suggest that corrupt behavior in the task was extremely rare. However, one must take into account that bribes were offered only in 20% of all trials and only in two-third of these trials was the classification according to shape different from the classification according to color. From the trials with misaligned classification criteria, participants took the offered bribe and harmed the charity in approximately 15% of cases on average. Nevertheless, almost 40% of participants did not take any bribe that would harm the charity.

In line with our expectations, we found that taking bribes was associated with the size of a bribe, with the perception of the rule violation as not moral, and with lower scores on the honesty-humility scale from the HEXACO personality inventory.

The correlation with the honesty-humility scale (and an absence of any correlation with the other five personality traits) suggests that our task is in fact associated with honesty and not with the related concepts such as agreeableness (Becker et al., 2012). The honesty-humility scale has been previously found to be associated with cheating (Hilbig and Zettler, 2015) and self-reported workplace delinquency (Lee et al., 2005). The present study further shows the validity of the honesty-humility scale in predicting moral behavior.

In the present study, women were somewhat less likely to take bribes than men. The results seem to support general proclivity for less corrupt behavior in women as described by Chaudhuri (2012). However, similarly as most of the past research, the present study used a convenience sample, which limits any generalization of the gender effects, and should be therefore interpreted with caution, especially because other studies suggest that the observed gender differences may be caused by the differences in beliefs about the prevalence of corrupt behavior between women and men (Shaw et al., 2013; Fišar et al., 2016).

Trial-level analysis showed that response times were longer for trials with bribes and even longer for trials with bribes that were accepted, suggesting that acting corruptly requires overcoming an automatic honest response. It could be argued that the trials with bribes were less frequent than the trials without bribes, and hence the longer response times might have been simply associated with a higher cognitive load. Since participants mostly classified the objects according to their color, it is possible that classifying an object according to its shape to take a bribe was less automatic, and thus resulted in longer response times. This possibility does not, however, fully explain the effect of the bribe size on response times. Participants took longer to decide whether to take bribes of higher values, regardless of whether they took the bribe or not. This suggests that the decision concerning larger bribes was not just less automatic, but it was also associated with a stronger motivational conflict. This result is in line with previous findings that identify approximately 40% of participants as unconditionally honest, 25% as unconditional cheaters, and the rest as susceptible to the situational factors (Fischbacher and Föllmi-Heusi, 2013; Rosenbaum et al., 2014). While the participants who are not influenced by the bribe size in their decision to take it or not probably do not show the effect on response times, large bribes may be tempting even for those who have reservations about breaking the rules and causing harm and therefore experience a stronger conflict when deciding whether to act dishonestly or not.

We found no support for the moral licensing hypothesis in the present study. However, as can be seen from **Figure 3**, our assumption that participants would be less likely to take smaller bribes in the low bribe condition was erroneous; smaller bribes in both groups were taken with similar probabilities. Therefore, our experimental manipulation failed to create conditions in which the moral licensing effect would be expected to occur.

### Limitations and Future Directions

In its current implementation, the game focuses solely on the behavior of a bribee, not a briber. There is, therefore, no interaction between a potential briber and the bribee, and participants are not told from whom they receive the bribes. This is comparable to many real-life cases of corruption where a public official, who for example issues permits or conducts inspections, is offered bribes in exchange for making speedy favorable decisions or ignoring non-compliance. The public official in these instances has only a very limited interaction with the bribers and sometimes does not need to interact with them directly at all. This understandably differs from the situation where an official has repeated interactions with the briber, such as in a relationship of a customs officer or a politician with a company for which they repeatedly provide illegal benefits. In the latter case, the outcome of the briber as well as their mutual trust with the official may play a role in the bribee’s decision-making, which could be therefore better modeled with a task in which these factors are present (e.g., Weisel and Shalvi, 2015).

That bribes were not offered by another person, but were generated randomly,^8^ is just one of a variety of features of the game that can be adjusted for particular experimental needs in its different implementations. Other such examples are that bribes and corruption were not explicitly mentioned, and the third party harmed by the incorrect performance of the task was a charitable organization and participants knew exactly how much harm they had caused it. Or that the sorting rule in the present implementation of the game allowed obtaining bribes even in cases when the sorting rule was not violated (i.e., when the classifications according to color and shape were aligned). All these details are purely accidental and could be easily changed in the future. For example, different third parties could be used, taking a bribe could always be associated with a rule violation, and bribes could be sent by other participants. The task might be also adjusted to share more features with corruption in the real world by, for example, adding the possibility of detection and punishment of corrupt behavior. Its external validity for studying corruption can thus be further improved.

## Conclusion

We introduced a new experimental task for the laboratory study of decision to accept bribes. The task includes a norm violation that is absent in most of the previously used tasks. Using the new task, we found that bribes, especially larger ones, are tempting even for those who do not take them at the end – as shown by longer reaction times not only on trials with bribes present in general, but also on those in which bribes were not accepted. These results are in line with previous findings that identified a substantial proportion of conditional cheaters among participants of various experiments. People belonging to this group should be of primary interest to any potential anticorruption interventions (Houdek, 2017). Furthermore, our findings also suggest that personality scales, namely the honesty-humility subscale of the HEXACO inventory, could be useful for identifying persons with such higher propensity for taking bribes and acting corruptly.

## Ethics Statement

This study was carried out in accordance with the recommendations of “Ethical Principles of Psychologists and Code of Conduct by American Psychological Association” with informed consent from all subjects. The study does not involve vulnerable populations, poses no risk for participants and uses no deception. All subjects gave informed consent in accordance with the Declaration of Helsinki. In accordance with Czech laws and institutional regulations of the University where the study took place, the protocol was exempt from the approval process by the IRB and it was not necessary to obtain written consent from the subjects.

## Author Contributions

Both authors designed the study and wrote the manuscript. ŠB coded the experimental task and analyzed the data. MV collected the data.

## Conflict of Interest Statement

The authors declare that the research was conducted in the absence of any commercial or financial relationships that could be construed as a potential conflict of interest.

## Footnotes

1 Results for a test of an additional preregistered hypothesis concerning the charity incurring a loss are not reported here for the sake of brevity and can be found at https://osf.io/ak8un/.

2 Partial correlations controlling for the low bribe and high bribe conditions yielded virtually the same estimates in all participant-level analyses. Earnings for oneself were correlated somewhat more strongly with sincerity (*r*_S_ = −0.24) and greed avoidance (*r*_S_ = −0.20) subscales than with fairness (*r*_S_ = −0.14) and modesty (*r*_S_ = −0.14). The proportion of bribes taken was correlated somewhat more strongly with fairness (*r*_S_ = −0.16), greed avoidance (*r*_S_ = −0.12), and sincerity (*r*_S_ = −0.11) subscales than with modesty (*r*_S_ = −0.04).

3 The honesty-humility scale contains an item specifically asking about taking a bribe (“I would never accept a bribe, even if it were very large”). When honesty-humility is analyzed without this question, it still predicts participants’ earnings (*r*_S_ = −0.25, 95% CI = [−0.38, −0.12], *p* < 0.001), but the association with the proportion of bribes taken is no longer significant (*r*_S_ = −0.14, 95% CI = [−0.27, 0.01], *p* = 0.05). The item itself is not significantly associated with participants’ earnings (*r*_S_ = −0.07, 95% CI = [−0.21, 0.09], *p* = 0.36), or the proportion of bribes taken (*r*_S_ = −0.12, 95% CI = [−0.26, 0.01], *p* = 0.09).

4 The estimates can be interpreted as effects of the increasing from the minimum value of a given variable to the maximum.

5 OR – odds ratio.

6 Including random slopes for bribe sizes also did not significantly improve the base model without any random slopes.

7 The reaction times depend partly on a momentary state of a participant. For example, if participants do not pay much attention for a while, this will result in slower reaction times for several trials. The reaction times of subsequent trials are therefore usually correlated with each other more than with trials that are further away. Inclusion of reaction times on preceding trials is used to remove the resulting autocorrelation of residuals. The reported analyses yield virtually the same results as the analyses without inclusion of the reaction times for preceding trials as covariates. The only exception are the effects of trial order and charity earnings at the point of the decision, which are both themselves autocorrelated and are therefore reduced when the additional covariates are included.

8 However, the participants were not informed of the nature of the bribes’ origin.
